# Predicting sedimentary bedrock subsurface weathering fronts and weathering rates

**DOI:** 10.1038/s41598-019-53205-2

**Published:** 2019-11-20

**Authors:** Jiamin Wan, Tetsu K. Tokunaga, Kenneth H. Williams, Wenming Dong, Wendy Brown, Amanda N. Henderson, Alexander W. Newman, Susan S. Hubbard

**Affiliations:** 1 Earth and Environmental Sciences Area, Lawrence Berkeley National Laboratory, Berkeley, California USA; 2 Rocky Mountain Biological Laboratory, Crested Butte, Gothic, Colorado, USA

**Keywords:** Element cycles, Hydrology

## Abstract

Although bedrock weathering strongly influences water quality and global carbon and nitrogen budgets, the weathering depths and rates within subsurface are not well understood nor predictable. Determination of both porewater chemistry and subsurface water flow are needed in order to develop more complete understanding and obtain weathering rates. In a long-term field study, we applied a multiphase approach along a mountainous watershed hillslope transect underlain by marine shale. Here we report three findings. First, the deepest extent of the water table determines the weathering front, and the range of annually water table oscillations determines the thickness of the weathering zone. Below the lowest water table, permanently water-saturated bedrock remains reducing, preventing deeper pyrite oxidation. Secondly, carbonate minerals and potentially rock organic matter share the same weathering front depth with pyrite, contrary to models where weathering fronts are stratified. Thirdly, the measurements-based weathering rates from subsurface shale are high, amounting to base cation exports of about 70 kmol_c_ ha^−1^ y^−1^, yet consistent with weathering of marine shale. Finally, by integrating geochemical and hydrological data we present a new conceptual model that can be applied in other settings to predict weathering and water quality responses to climate change.

## Introduction

Chemical weathering impacts the global CO_2_ budget, atmospheric oxygen uptake, and nutrients and metals cycling in ecohydrological systems^1–3^. Numerous models have been developed to estimate bedrock weathering impacts on global elemental cycling and water quality based on relative rates of land surface erosion and downward propagation of weathering fronts^4–7^. Hydrological processes exert important controls on weathering depths^8–12^. The most dominate weathering reactions within sedimentary rocks are pyrite oxidation and carbonate (calcite and dolomite) dissolution^13–17^. Water and atmospheric oxygen together drive pyrite oxidation and produce sulfuric acid, which in turn drives carbonate dissolution. Oxidation of organic matter yields carbonic acid that also drives carbonate dissolution. The other common minerals in sedimentary rocks sensitive to proton concentration include clay minerals such as chlorite and illite^15,17–19^. Oxidation of rock-organic carbon (OC) is another important weathering reaction^3,20^. The common understanding is that, as the most reactive mineral, pyrite has the deepest weathering front and the other minerals’ weathering fronts are stratified (nested) into shallower depth intervals^8,9,17,21^. Despite these significant advances, the actual depths of weathering reactions in the subsurface are often unknown, so that predicting weathering depths associated geochemical exports remains challenging. One of the major barriers is that the identification of a weathering front relies on quantifying individual mineral’s concentration profile relative to its parent rock, while the porewater chemistry associated with weathering rocks is seldom available. Pore water chemistry is often inferred from sampling nearby seeps, streams, and rivers, yet such waters lack needed spatial, temporal, and hydrologic information. Additionally, to the best of our knowledge, the current understanding of weathering fronts lacks insights into hydrologic controls obtainable from measurements of hydraulic conductivities, hydraulic gradients, and seasonal water table dynamics. Therefore, quantification of subsurface weathering rates has been limited.

**Figure 1:**
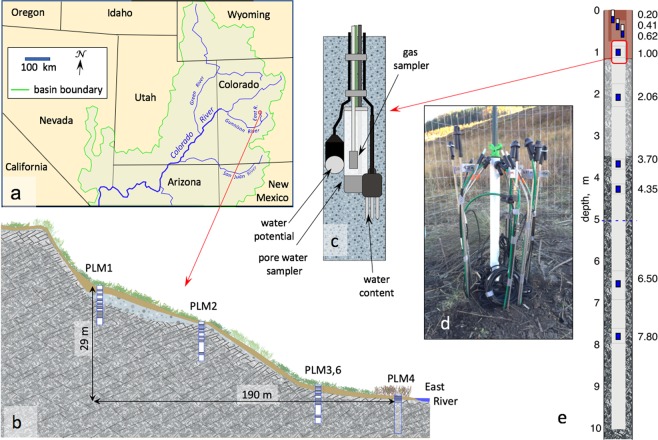
The East River hillslope-floodplain study site and instrumentation. (**a**) The upper Colorado River Basin and the location of the East River watershed hillslope site (red circle). (**b**) Sampling and instrumentation locations along the hillslope-floodplain transect. Stations PLM3 and PLM6 are at the same elevation, separated by 4.5 m. PLM6 was drilled to install a groundwater monitoring well, but does not contain depth-distributed instrumentation. At station PLM4, samplers and instruments were installed to a maximum depth of 1.3 m due to borehole collapsed. Note that PLM2 is locally elevated by ~2.5 m relative to the overall transect slope. (**c**) Specific depths within boreholes were instrumented with pore water samplers, water content sensors, water potential sensors, and gas samplers. (**d**) Outlets of samplers and sensors at the soil surface. (**e**) Example of depth distribution of instruments within a borehole (PLM3).

The East River study site, is a headwater catchment in the Upper Colorado River Basin (Fig. 1a). Weathering of the underlying Mancos Shale and similar marine shale bedrock found throughout the southwestern United States is known to contributes to elevated concentrations of major ions, trace elements, and potential contaminants^22–25^. Shale weathering significantly contributes to the East River’s seasonally varying water chemical composition^21,26^. Along a 190 m hillslope to floodplain transect, we drilled five 10 m deep boreholes into the parent rock (Fig. 1b), with four along the hillslope and one on the floodplain for soil and rock analyses, and to install instruments for long-term hydrologic and geochemical monitoring^27,28^ (Fig. 1c–e). The results are presented in five parts: (1) soil to bedrock elemental and mineral composition constraints on weathering fronts; (2) soil to bedrock porewater chemical composition constraints on weathering fronts; (3) seasonal water table depth relationships with weathering fronts; (4) measurements-based subsurface weathering rates; and (5) a new conceptual model that couples subsurface weathering fronts to water table positions, and suggests that pyrite, carbonates and fossil organic matter (OM) share the same weathering front in sedimentary bedrock.

## Results and Discussion

### Soil to bedrock elemental and mineral compositions marked weathering fronts

**Figure 2:**
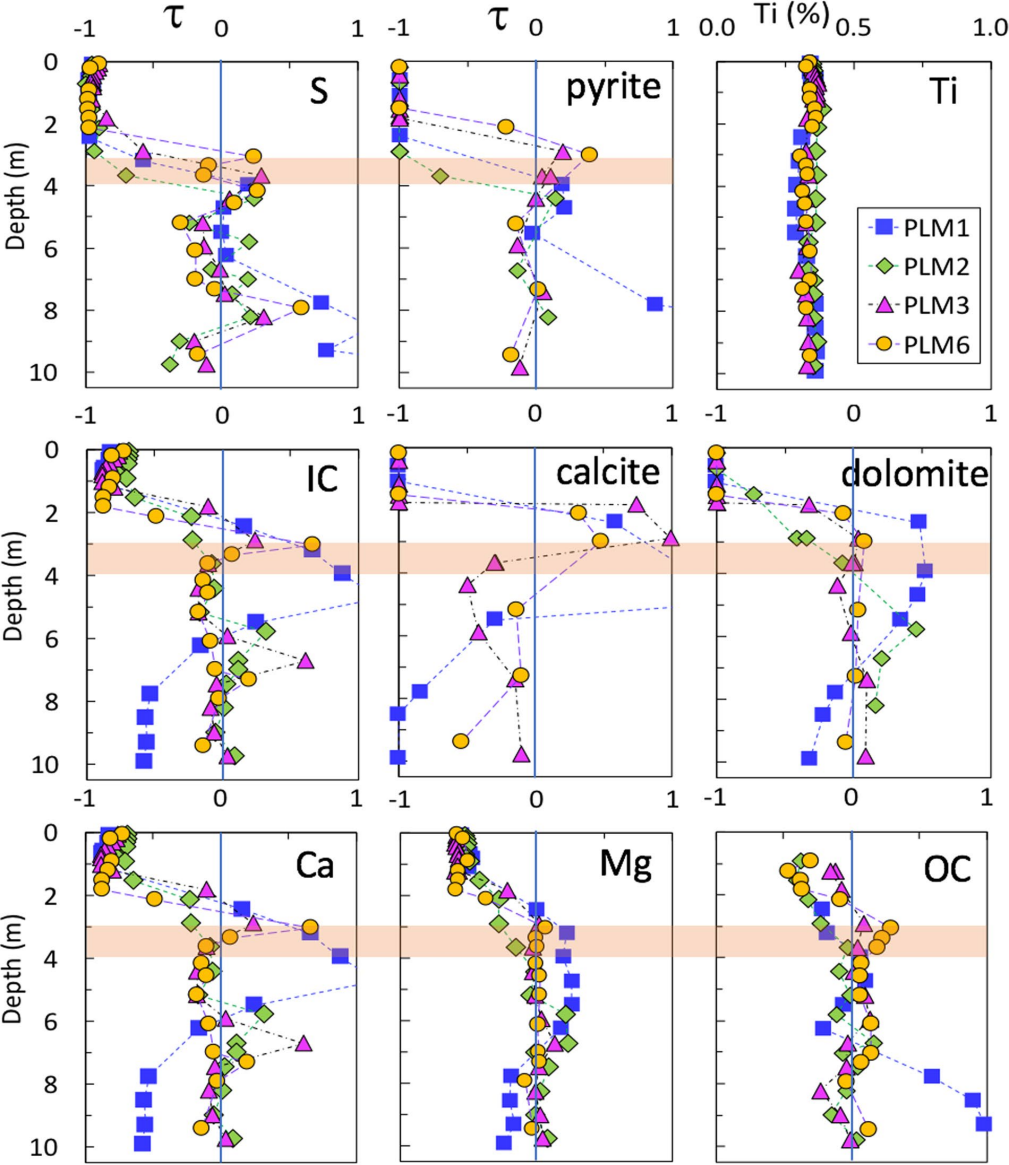
Relative concentrations *τ*_i,j_ of the most weathering relevant elements and minerals. Titanium (upper right graph) is used as the “immobile element *i*” in the calculations. The vertical lines at *τ*_*j*_ = 0 separates the concentration values into losses (*τ*_*j*_ < 0) and gains (*τ*_*j*_ > 0) relative to the parent rock. *τ*_*j*_ = −1 indicates complete loss of *j*. Here a weathering zone (WZ) is defined as the interval between *τ*_*j*_ = −1 and 0, and weathering front is at *τ*_*j*_ = 0 (where *τ*_*j*_ value crossing the vertical line). The horizontal orange band marks the depth range of weathering fronts of the four PLM locations along the hillslope flow transect.

We operationally defined the thickness of the soil profile by the depth at which manual core sampling was no longer possible (Fig. S1a). Based on 40 hand-augered soil cores, the soil depths are 0.9–1.1 m, 0.7–0.9 m, and 1.0–1.3 m below ground surface (bgs) at stations PLM 1, 2, and 3, respectively. Thus, the soil depths on this hillslope are approximately 1.0 ± 0.3 m bgs. At PLM4, the soil depth is 0.7–0.8 m bgs. The PLM4 floodplain station is less discussed later because this paper is focused on hillslope weathering. Distinct, progressive changes in color and texture of the rock core samples were observed with depth (Fig. S1b), and the contrasting characteristics qualitatively distinguish three zones: soil, weathering zone (WZ), and unweathered fractured bedrock (FBR) or parent rock. The WZ refers to the depth interval were the primary weathering reactions occur, and we refer to the lower boundary of a WZ as its weathering front. Based on the analyses of five cores, the unweathered shale bedrock contains: 1.1 ± 0.5% S, 3.2 ± 0.5% Fe, 1.31 ± 0.15% organic carbon (OC), 1.4 ± 0.5% inorganic carbon (IC), 3.5 ± 1.5% Ca, 1.7 ± 0.2% Mg, 2.3 ± 0.2% K, and 0.62 ± 0.08 Na (Fig. S2). The parent rock contains 3.0 ± 1.0% pyrite, 4.6 ± 2.9% calcite, 7.5 ± 1.5% dolomite, 2.4 ± 0.6% chlorite, 30 ± 4% illite (layered with smectite or muscovite), 8.5 ± 2.4% plagioclase, and 45 ± 4% quartz. Relative concentrations *τ*_*i,j*_ ^29,30^, defined by Eq. (1), are used to evaluate weathering caused losses and gains of the elements and minerals relative to the parent rock,

1 $${\tau }_{i,j}=\frac{{C}_{j,w}{C}_{i,p}}{{C}_{j,p}{C}_{i,w}}-1$$

where *C* is concentration, the subscript *j* represents a mobile constituent (element or mineral of interest), and subscript *i* represents the selected immobile reference element associated with the parent rock. In the calculations we used titanium (Ti)^30^ as the immobile element *i*. The subscripts *w* and *p* denote weathered and parent rock, respectively. The values of element/mineral *j* and *i* in parent rock were determined by averaging the data of parent rock. The selected relative concentrations *τ*_*j*_ for the elements and minerals most diagnostic of weathering are presented in Fig. 2. Following convention, the WZ is defined as the depth range between *τ*_*j*_ = −1 (complete depletion) and *τ*_*j*_ = 0 (the weathering front). It is important to note that the precision with which the weathering front position can be identified is limited by the depth increments used for sampling.

Figure 2 shows that carbonate and pyrite are nearly completely depleted (τ_j_ = −1) from the surface down to about 2.0 to 3.0 m bgs, respectively. In this figure the orange bands are used to mark the depth range of weathering fronts at *τ*_*j*_ = 0. The τ-sulfur values of the hillslope locations cross the vertical zero line at depths of about 4.0 m bgs, and slightly deeper for PLM2. Lacking other sulfur-containing minerals at significant concentrations at our site, the τ plots of sulfur (analyzed relatively easily and accurately by XRF) are more practical for identifying pyrite weathering fronts than the τ plots of pyrite, because quantitative mineralogical analysis is more costly. Trace level of hematite, one of the eventual products of pyrite oxidation, occurred at low levels (≤0.5%, and up to 0.9% for two samples) within the WZ, and was undetected in the parent rock. Relative to pyrite, it is difficult to precisely locate the carbonate weathering front depths, because of cyclic dissolution and reprecipitation of calcite, shown by the τ values of inorganic carbon and calcite within the depths 2.0 to 4.0 m bgs. Note that PLM2 borehole samples lack calcite but contain dolomite, while PLM1 is enrich in dolomite in the 2.5–4.5 m bgs interval. Compared to pyrite, the highly solubility-sensitive nature and the heterogeneous distribution in parent rocks obscured the delineation of carbonate weathering fronts. Despite these limitations, the data suggest carbonate weathering fronts reside in the range of 2.5 to 4.0 m bgs. Soil organic carbon (SOC) concentrations are as high as 8.5 mass% within the shallow soil, and decrease rapidly with the depth to a sharp transition at ~1.0 m bgs from SOC to rock-OC (Fig. S2). Hence in Fig. 2, the τ-OC plot starts from 1.0 m bgs down. Rock-OC depletion is reflected by the τ-OC values declining to −0.5 (50% of the rock-OC depletion) at about 1.0 m bgs, the bottom of soil. The τ-OC values cross zero at roughly the same depth range as pyrite and carbonate, 3.0 to 4.0 m bgs. Thus, within the resolution of our sampling, the weathering fronts for pyrite, carbonate minerals, and rock-OC are overlapping.

### Soil to bedrock porewater chemical composition delineated weathering fronts

**Figure 3:**
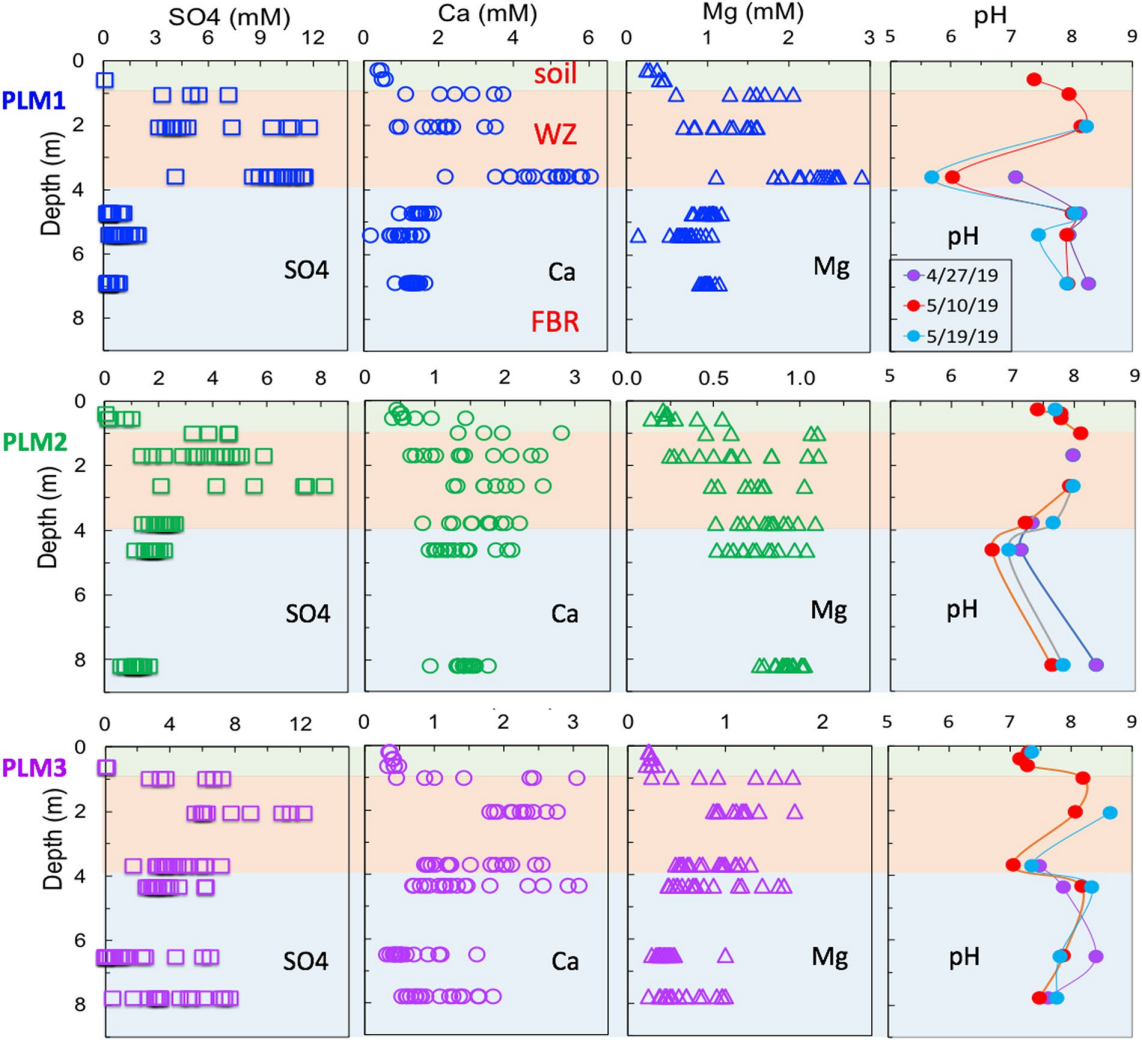
Depth profiles of pore water SO_4_, Ca, Mg, and proton concentrations. Most of the pore water samples were collected from October 2016 to May 2019, but the samples for pH were only from spring 2019. Approximate ranges of three vertical zones: soil, WZ (weathering zone), and FBR (fractured bedrock), are marked with green, orange and blue background, respectively. The pore water SO_4_^2−^ concentration profiles result from pyrite dissolution. The Ca and Mg profiles result mainly from dissolution of carbonate minerals. The pH profiles reflect the effects of proton generation by pyrite dissolution with additional contributions of organic carbon oxidation, and neutralization by carbonate dissolution.

**Figure 4:**
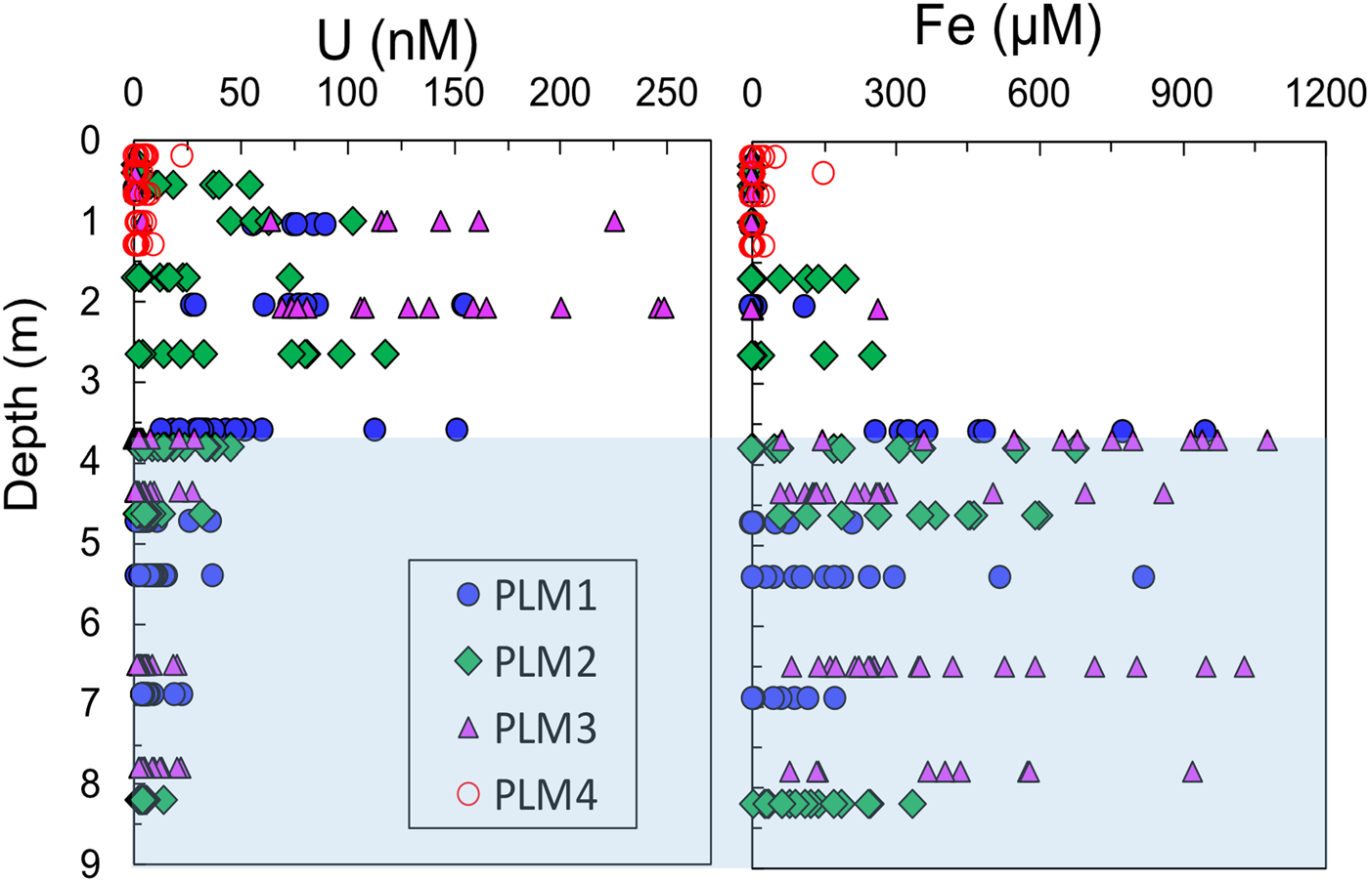
Depth profiles of U^6+^ and Fe^2+^ concentrations. The data show the redox boundary at about 4.0 m bgs for the hillslope locations (the shaded region is the reducing zone). The data support the weathering front locations identified by the τ plots and porewater SO_4_^2−^ concentration-depth profiles.

As previously noted, the precision with which weathering fronts can be identified is limited by the depth increments associated with sampling, and the following results are presented with this limitation in mind. The three zones (soil, WZ, FBR) identified in the previous section are marked on Fig. 3 by background colors of green, orange and blue, respectively. Figure 3 shows the depth profiles of selected chemical components in pore waters from the same boreholes where the solid samples were taken. The porewater samples were collected into suction lysimeters installed as deep as 8.20 m bgs at PLM 1, 2 and 3, from October 2016 through May 2019, approximately monthly (except during freezing weather and when the soils were too dry). The samples for pH measurement were collected in Spring 2019 only. To the best of our knowledge, such *in-situ* solute concentration profiles through weathering bedrock were not previously available, yet are informative for identifying weathering reactions and their depth distributions. The production of SO_4_^2−^ (the three graphs along the first column of Fig. 3) results directly from pyrite oxidation-dissolution. The elevated SO_4_^2−^ concentrations are situated in the depths consistent with that of the τ plots identified WZ depths of ~4.0 m bgs. Annual snowmelt recharge drives SO_4_^2−^ and other weathering products downslope along the weathering zone as well as downward into bedrock. In the FBR below the WZ (depths greater than 4.0 m bgs), PLM1 and PLM2 show low and nearly constant SO_4_^2−^ concentrations (n = ~30 at these deep locations), while high and fluctuating SO_4_^2−^ concentrations are observed in the FBR at PLM3. The highly variable SO_4_^2−^ concentrations at deeper PLM3 suggest recharging pore waters flowing through the weathering zone traverse through the complex fractured rock pathways. Lack of pyrite dissolution at depths below 4.0 m is indicated not only by the values of τ-sulfur = 0 and τ-pyrite = 0 at ~4.0 m bgs for PLM3 (Fig. 2), but also by the redox boundary at about 4.0 m bgs delineated by the concentration profiles of redox-sensitive Fe^2+^ and U^6+^ (Fig. 4). Thus, pyrite oxidation is prohibited in the reduced FBR zone.

The pore water Ca^2+^ and Mg^2+^ profiles (graphs in the two middle columns of Fig. 3) are the products of carbonate mineral weathering-dissolution. Note that their concentration-enriched depths are the same as the that of pyrite for PLM1, and deeper than that of pyrite for PLM 2 and 3, contrary to the understanding that the carbonate weathering front is nested at a shallower depth then pyrite. Like our solid phase analyses, these new porewater concentration depth profiles challenge the previous understanding based mainly on solid phase τ values. The data show that carbonate weathering front is at (PLM1), or deeper (PLM2 and PLM3) than that of pyrite. The acid generated from pyrite dissolution and resulting increased rock permeability together drive carbonate dissolution. Thus, acidic dissolution of carbonate minerals occurs down to at least the pyrite weathering front. The favorable oxygen and moisture conditions for pyrite oxidation at the weathering front also stimulate rock organic matter degradation, as suggested by the elevated DOC concentrations in the WZ pore waters (data are not shown), up to 50% depletion of the rock-OC, and the τ-OC decreasing below zero at locations as deep as ~4.0 m bgs (Fig. 2). The rock permeability and hydraulic gradient below the weathering zone control whether or not carbonate dissolution goes beyond the depths of the pyrite weathering front, because the generated acid is advectively transported. This analysis is supported by the depth profiles of pore water pH from recently collected samples (the graphs on the right column in Fig. 3). The pH profiles reflect the combined effects of proton generation by pyrite dissolution and rock organic carbon oxidation, and neutralization by carbonate dissolution. Acid generated during pyrite oxidation not only drives carbonate dissolution at the same depths as that of pyrite weathering front (PLM1), but also slightly deeper (PLM 2, 3). Similarly, Chigira^15^ previously showed that in mudstone bedrock of a mountainous watershed in Japan, the chlorite dissolution zone was below the pyrite oxidation front due to migration of sulfuric acid generated at the oxidation front. In summary, the porewater chemical profiles suggest that carbonate minerals and potentially other rock components share the same weathering front depth with pyrite, and are not spatially stratified as previously thought. Reaction rates only control the time scale for depletion completion of individual minerals, but do not determine the depth of reaction front.

### Seasonal water table depths and the relationship with weathering fronts

**Figure 5:**
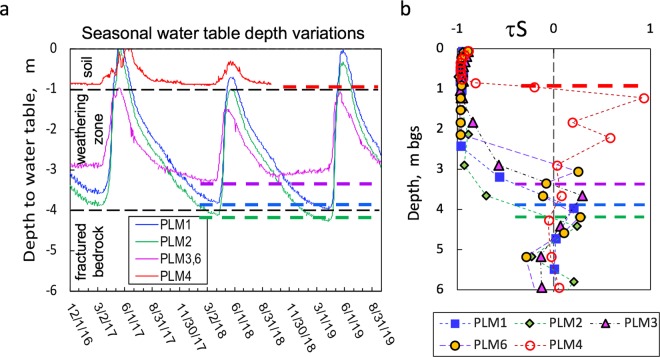
Measured seasonal water table depths and relations with weathering fronts at four locations along the hillslope flow transect. (**a**) Data of seasonally fluctuating water tables from four borehole locations, showing that the water table rises and falls synchronously along the hillslope, and the lowest water table depths in the 2018 are marked by the dashed-lines. The greater depth of the water table at PLM2 reflects the fact that its soil surface is elevated relative to the mean slope between PLM1 and PLM3,6. (**b**) The weathering fronts defined by τ-S, τ-pyrite and porewater SO_4_^2−^ concentration profiles (marked by the dashed-lines) consistent with the lowest seasonal water table depths.

The measured water table depths at the four locations along the hillslope flow transect from winter 2016 through summer 2019 are plotted in Fig. 5a. The steep rises result from annual snowmelt, the main source of groundwater recharge. Because the snowpack at the study site in 2017 and 2018 was exceptionally high and exceptionally low, respectively, the water table trends span expected extremes with respect to their ranges of depths. The measured deepest water tables from the 4 locations indicated by the horizontal dashed lines are at 3.9, 4.2, 3.3 m bgs for sites PLM1, 2, 3, respectively, and 0.9 m bgs for the floodplain site PLM4 (Fig. 5a). In Fig. 5b, the pyrite weathering front depths for the same locations are shown (marked by the same colored dashed-lines), collectively determined from τ-S and τ-pyrite, and porewater SO_4_^2−^ concentration profiles (Figs 2 and 3). Comparison of these weathering front depths in the τ-S plots with the baseflow water table levels shows good agreement between the annual maximum depths of the water table and the depth below which pyrite oxidation becomes negligible. Thus, these findings support identifying the lower boundary of the weathering zone with the maximum depth of the water table. These findings on the position of the pyrite oxidation front along the hillslope-floodplain transect are consistent with the model of Winnick *et al*.^21^, and also with identification of pyrite oxidation at a depth of 23 m bgs at a ridge in the Susquehanna Shale Hills Observatory by Brantley *et al.*^8^. There, the annual water table fluctuated between 19 and 26 m bgs. It is worth noting that, unlike our findings, analyses of fractured metamorphic bedrock in the Upper Snake River basin revealed the presence of pyrite in samples up to 10 s of meters above the maximum water table elevation^10^. This disequilibrium is consistent with the generally slower weathering of metamorphic rocks relative to sedimentary rocks^1^.

### Subsurface measurements-based weathering rates

**Figure 6:**
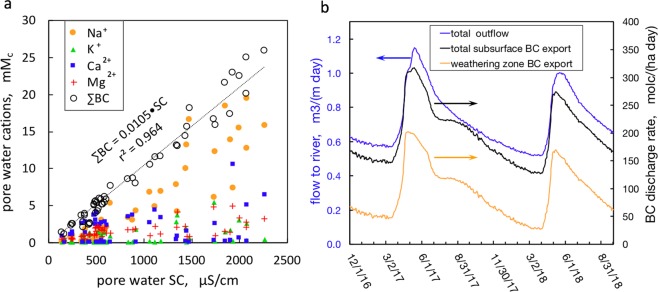
Calculating hillslope base cation export rates. (**a**) Correlations between specific conductance, individual base cations, and sum of base cations (∑BC) in hillslope pore waters. **(b)** Daily subsurface flow and export rates of base cations. The calculated net annual exports by summing the daily discharge rates are 75 and 67 kmol_c_ ha^−1^ y^−1^ for WY2017 and WY2018, respectively.

Having constrained the depth interval over which active weathering occurs and solutes from weathering are generated, information on concentrations of major ions and subsurface flow rates needed to be integrated to estimate weathering rates. As shown recently^25^, flow within the weathering zone of the lower hillslope has a persistent component directed downslope, parallel to the topography, rather than directly downward into the fractured bedrock. This downslope weathering zone flux is periodic, becoming maximum during snowmelt, and minimum just prior snowmelt when the water table has receded to the weathering front. In that companion hydrologic study^25^, the transmissivity feedback method^31^ was used to estimate the depth- and time-dependence of subsurface flow and transport of major ions (as specific conductance, SC) on the hillslope. Given the practically direct proportionality between SC and the sum of base cations (BC including Na^+^, K^+^, Ca^2+^, and Mg^2+^) shown in Fig. 6a, results from the hydrologic analysis were applied to calculate hillslope weathering in terms of rates of BC depletion. The calculated hillslope subsurface flow to the East River, and associated BC discharge rates are presented in Fig. 6b. The daily volumetric flow rates normalized per unit meter width of the transect during water year (WY) 2017 to WY2018 (depicted as the blue trend in Fig. 6b) were presented in the recent hydrologic analysis^25^, and reflect the importance of spring snowmelt. Because continuous groundwater elevation measurements began at the end of November 2016, WY2017 was defined as the interval from 12-1-2016 to 11-30-2017, rather than the conventional interval beginning on October 1 and ending on September 30. Daily rates of BC discharging from the total subsurface and discharging along the weathering zone are respectively shown in the black and orange trends in Fig. 6b. Note that these export rates rise during snowmelt because water table rise into the weathering zone drives downslope flow of the solute-rich pore waters. In order to compare the overall weathering rates on this hillslope with values reported from other regions, the net annual export of BCs was calculated by summing the daily discharge rates (Fig. 6b) for WY2017 and WY2018, yielding 75 and 67 kmol_c_ ha^−1^ y^−1^, respectively. These weathering rates are very high relative to annual BC exports from watersheds underlain by igneous and metamorphic rocks^32,33^, and slightly higher than the broad range (3 to 66 kmol_c_ ha^−1^ y^−1^) reported from sedimentary rocks draining into the Yellowstone River^23,24,34^.

### Conceptual model

**Figure 7:**
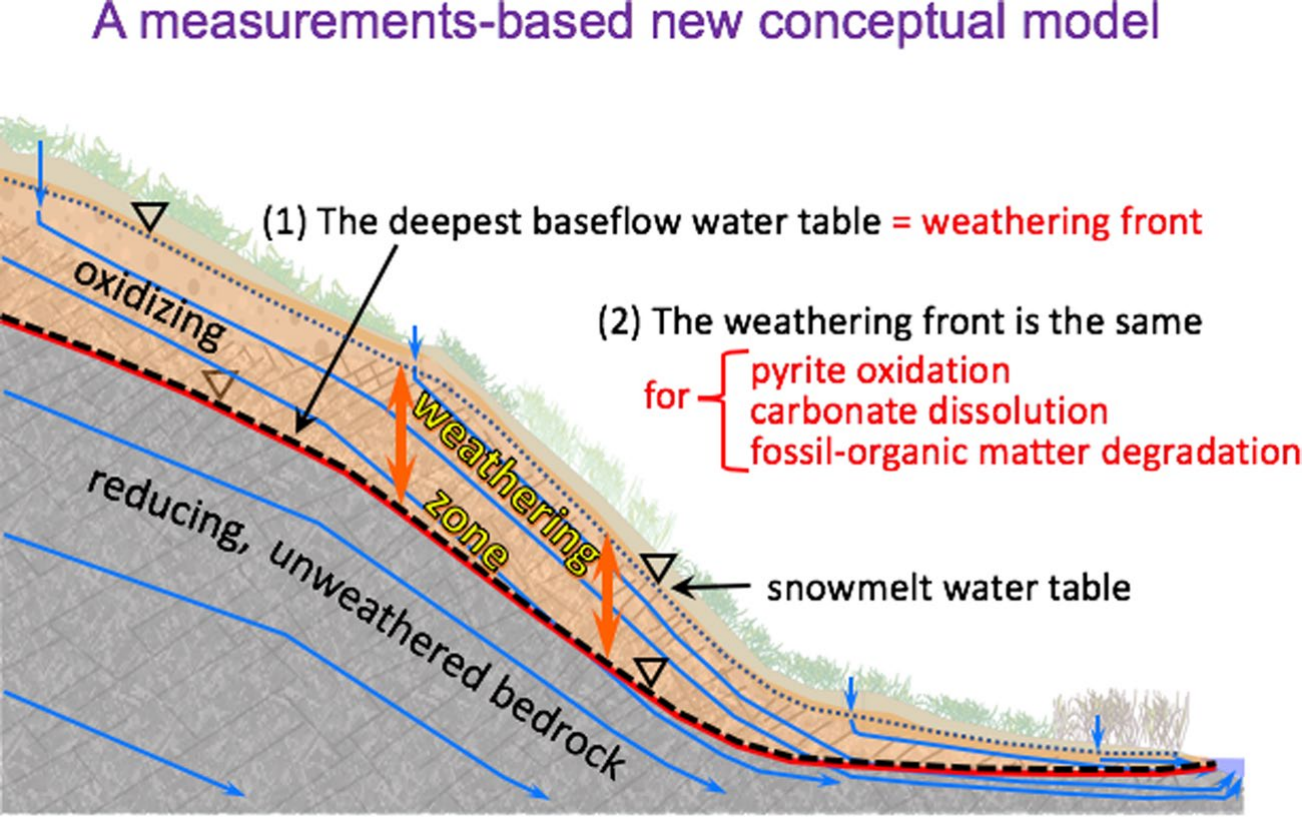
The new conceptual model couples subsurface water table positions with weathering fronts of multiple minerals and components in sedimentary bedrocks. The water table remains approximately parallel to the soil surface along the hillslope. The deepest seasonal extent (the minimum baseflow water table) determines the pyrite weathering front. Carbonate minerals and fossil organic matter share the same weathering front with pyrite.

The new conceptual model (Fig. 7) couples subsurface water table positions with the weathering fronts of multiple minerals and components in sedimentary bedrocks. In this model the pyrite weathering front depth is predictably located at the deepest water table depth of annual water table oscillation. The active weathering zone is located in between the shallowest snowmelt water table and the deepest baseflow water table. This deepest baseflow water table divides the subsurface into variably aerated soil and weathering zone, and permanently reducing fractured bedrock zone. Carbonates and rock organic matter share the same weathering front with pyrite. Snowmelt recharge drives the acidity generated from pyrite oxidation downward, and therefore must drive dissolution of carbonate minerals at and below the pyrite weathering front. Reaction rates only control the time scale for depletion of individual minerals, but do not determine the depths of reaction fronts. While rates of weathering of these latter components are slower than that of pyrite, increased oxygen availability, acidity, and permeability at the pyrite weathering front act on all of these substrates. This new measurements-based conceptual model developed through the inclusion of pore water chemistry above and below the water table, in combination with continuously monitored water table depths provides a refined understanding of subsurface bedrock weathering, and a framework and an approach for predicting spatial and temporal variations in solute exports to surface waters.

## Methods

### The study site

The field study site is in East River Watershed, a headwaters catchment located in the Upper Colorado River Basin, 5.3 km south-southwest of Gothic, Colorado (Fig. 1a). The East River discharges into the Gunnison River and in turn to the Colorado River. The upper East River watershed has a mean annual precipitation of about 700 mm, with about 70% occurring as snow occurring from October to May, although decreased snowpack and earlier snowmelt are resulting from climate warming. Additional precipitation is received as summer monsoonal rain. The study site is in an area representative of the watershed’s lower montane hillslope and floodplain environments. Vegetation on the lower montane hillslope consists of grasses, shrubs, and forbs, and the floodplain adjacent to the East River is vegetated with grasses, forbs, and shrubs (willows). The lithology of the study site and much of the region is predominantly Cretaceous Mancos Shale. Chemical weathering of the broadly distributed Mancos Shale contributes to elevated concentrations of major ions, trace elements, and potential contaminants throughout the southwestern United States^23,24^. Shale weathering fluxes significantly contribute to the East River’s seasonally varying water chemical compositions^21,26^.

### Instrumentation and hydraulic measurements

Along a 190 m hillslope to floodplain transect, five 10 m deep boreholes were drilled into the parent rock with four along the hillslope and one on the floodplain to collect soil and rock samples, and to install instruments for long-term hydrologic and geochemical monitoring (Fig. 1b). Stations PLM1, 2, and 3 on the hillslope were instrumented for depth-resolved measurements down to 8.2 m. Instruments included porewater samplers, gas samplers, pressure transducers, moisture sensors, and thermistors (Fig. 1c–e). At station PLM4, samplers and instruments were only installed to a maximum depth of 1.30 m due to borehole collapsed. Station PLM6, located 4.5 m away from PLM3 at the same elevation, was drilled to collect continuous core and install a groundwater monitoring well, but does not contain depth-distributed instrumentation.

The water table depth below the local soil surface at PLM1 and PLM6 was continuously recorded with pressure transducers (AquaTROLL 200). At all of the other PLM stations, water table depths were determined from equilibrium pressure measurements in porewater samplers using the “tensisampler” method and from depth-distributed moisture sensors^28^. At PLM4, depths to the water table were also obtained from the correlation between locally measured groundwater levels and continuously measured East River water levels.

### Soil, rock, and pore-water sampling and analyses

Rock samples were collected in 0.6 to 0.7 m depth increments from the dry-drilled boreholes at stations PLM1 through PLM4 (with a 0.14 m diameter ODEX bit with pneumatically ejected rock chips). Soil samples were collected near the locations of the boreholes with either a hand auger (0.07 m diameter) or with a slide-hammer coring tool (0.05 m diameter) in 0.10 m depth increments down to as deep as the hand auger could advance (typically to about 1.0 m bgs). Roots, only found in the shallow soil samples, were removed before further sample processing. Soil and the rock samples were oven dried at 75 °C for three days. The dry samples were powdered to less than ~50 µm for analyses. Elemental compositions were determined by X-ray fluorescence (http://www.alsglobal.com/geochemistry), and inorganic carbon (IC) and organic carbon (OC) in the solid samples were determined using a Shimadzu TOC-VCSH carbon analyzer. X-ray diffraction of soil/rock samples was conducted for quantitative mineralogical analyses (http://www.xrayminerals.co.uk).

Both vadose zone porewaters and groundwater were collected from the suction lysimeters installed at different depths at about monthly intervals. The collected waters were immediately filtered (0.45 µm polytetrafluoroethylene syringe filters), and then split into subsamples for cation (immediately acidified in the field and stored in a polyethylene vial), anion, and dissolved organic and inorganic carbon (glass vials with no headspace) analyses. The water samples in a cooler containing ice packs were shipped to the laboratory, and immediately stored in the refrigerator or frozen. Major and trace element cations were measured using an inductively coupled plasma mass spectrometry (Thermo Fisher, MA, USA), anions were measured using an ion chromatograph (ICS‐2100, Dionex, CA, USA), and dissolved carbon concentrations were determined with a TOC‐LCPN instrument (Shimadzu, MD, USA)^27^. The on-site and *ex-situ* porewater pH measurements were conducted in Spring 2019 only. The pH was measured immediately after the porewaters arrived to the laboratory. However, the porewater experiences degassing because of the suction applied to the sampler and later the pressure decreases, and therefore the overall measured pH values may be higher than the *in-situ* pH because of CO_2_ loss.

### Weathering rate calculations

Weathering rates are often quantified through losses of base cations (BC, the sum of Na^+^, K^+^, Ca^2+^, and Mg^2+^ equivalents) from watersheds^34^ (ref added). The specific conductance (SC) time trends reported in the companion hydrologic study^25^ were used to estimate the depth- and time-dependence of subsurface concentrations of base cations. The correlation between SC and BC was determined from a representative subset of pore water samples, then applied to the SC time trends to obtain BC time trends in pore waters. BC weathering rates are commonly normalized per unit area of watershed. Therefore, the product of flow rate times BC concentration was normalized on a daily basis to the area of the transect and its contributing upslope region to obtain the daily BC discharge rate. The integrated BC weathering rates were obtained by summing over 365 day intervals.

## Supplementary information

SUPPLEMENTARY INFO

## Data Availability

Data used in this paper are deposited in the U.S. DOE Environmental Systems Science Data Infrastructure for a Virtual Ecosystem (ESS-DIVE).
