# Supplementary material for: Predicting sedimentary bedrock subsurface weathering fronts and weathering rates
Source: Sci Rep. 2019 Nov 20;9:17198. doi: 10.1038/s41598-019-53205-2 (PMC6868182; doi:10.1038/s41598-019-53205-2)
Supplement: Supplementary file 1 — SUPPLEMENTARY INFO [file 41598_2019_53205_MOESM1_ESM.docx]

Predicting sedimentary bedrock subsurface weathering fronts and weathering rates

Jiamin Wan^1*^, Tetsu K. Tokunaga^1^, Kenneth H. Williams^1^, Wenming Dong^1^, Wendy Brown^2^, Amanda N. Henderson^2^, Alexander W. Newman^2^, Susan S. Hubbard^1^

^1^Earth and Environmental Sciences Area, Lawrence Berkeley National Laboratory, Berkeley, California, USA

^2^Rocky Mountain Biological Laboratory, Crested Butte, Gothic, Colorado, USA

**Contents of this file**

Figure S1

Figure S2


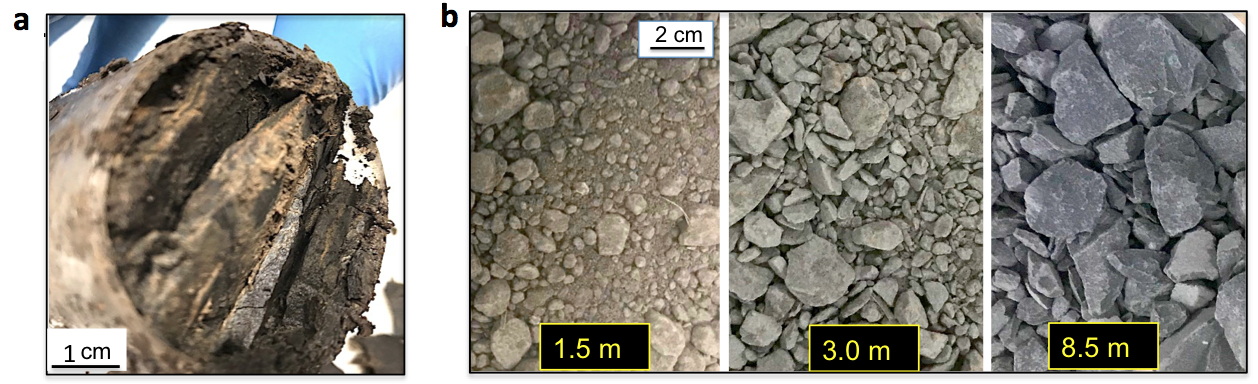


**Figure S1. Photographs of depth-resolved core samples showing different degrees of weathering. (a)** The bottom of a core (at 1.1 m below the ground surface) shows the depth limit for manual sampling occurs where weathering shale is encountered. **(b)** Examples of drill cuttings from different depths, showing progressive changes in rock color and structure. These samples are from PLM1.

**Figure S2. Depth profiles of selected major elements from boreholes along the hillslope flow transect.** Samples were analyzed using X-ray fluorescent (XRF) and Shimadzu TOC-VCSH carbon analyzer for IC and OC. PLM3 and PLM6 are at the same elevation and 4.5m apart. PLM4 is located at the edge of the floodplain (see map in Fig. 1b). For the OC% profiles, it should be noted that our radiocarbon age studies show that the ^14^C-OC Modern Fraction (MF) values rapidly change from 1.0 (modern OC only) to 0 (fossil OC only) going from 0.05 m in the soil surface to ~1.8 m bgs, and MF = 0 (fossil OC only) throughout the deeper rocks for all the profiles.
